# Tumor-Specific Delivery of Immune Checkpoint Inhibitors by Engineered AAV Vectors

**DOI:** 10.3389/fonc.2019.00052

**Published:** 2019-02-14

**Authors:** Johanna Reul, Janina Frisch, Christine E. Engeland, Frederic B. Thalheimer, Jessica Hartmann, Guy Ungerechts, Christian J. Buchholz

**Affiliations:** ^1^Molecular Biotechnology and Gene Therapy, Paul-Ehrlich-Institut, Langen, Germany; ^2^German Cancer Consortium (DKTK), Heidelberg, Germany; ^3^National Center for Tumor Diseases, Heidelberg, Germany

**Keywords:** antibody gene delivery, tumor targeting, checkpoint inhibition, biodistribution, adeno-associated virus

## Abstract

Immune checkpoint inhibitors (ICIs) can block distinct receptors on T cells or tumor cells thus preventing T cell inactivation and tumor immune escape. While the clinical response to treatment with ICIs in cancer patients is impressive, this therapy is often associated with a number of immune-related adverse events. There is therefore a need to explore innovative strategies of tumor-specific delivery of ICIs. Delivery of therapeutic proteins on a genetic level can be accomplished with viral vectors including those derived from adeno-associated virus (AAV). Here, we assessed the tumor-targeted Her2-AAV, a receptor-targeted AAV vector binding to the tumor antigen Her2/neu for cell entry, as vehicle for ICI gene delivery. Initially, we packaged the coding sequence of a scFv-Fc fusion protein directed against mouse programmed cell death protein-1 (PD-1) into Her2-AAV. Upon transduction of Her2/neu^+^ RENCA cells, AAV-encoded αPD-1 was readily detectable in the cell culture supernatant and revealed specific binding to its target antigen. *In vivo*, in BALB/c mice bearing subcutaneous RENCA-Her2/neu tumors, Her2-AAV mediated specific gene delivery into tumor tissue upon intravenous administration as verified by luciferase gene transfer and *in vivo* imaging thus demonstrating unimpaired tumor-targeting by Her2-AAV vectors in immunocompetent animals. When delivering the αPD-1 gene, levels of ICI were similar in tumor tissue for Her2-AAV and AAV2 but substantially reduced in liver for Her2-AAV. When combined with chemotherapy a tendency for reduced progression of tumor growth was documented for Her2-AAV treated mice. To get closer to the clinical situation, AAV constructs that deliver the complete coding sequence of the therapeutic antibody nivolumab which is directed against human PD-1 were generated next. The AAV-Nivolumab constructs were expressed and released from transduced MDA-MB-453 cells *in vitro* and from RENCA-Her2/neu cells upon intratumoral as well as intravenous administration *in vivo*. Antibody processing and expression levels were further improved through optimization of construct design. In conclusion, we provide proof-of-principle for redirecting the biodistribution of ICIs from liver and serum to tumor tissue by the use of engineered AAV vectors. This strategy can be easily combined with other types of immunotherapeutic concepts.

## Introduction

The paradigm of targeting exclusively the tumor cells during treatment of cancer patients is currently shifting toward strategies stimulating anti-tumor immunity. In fact, cancer immunotherapy has been celebrated as breakthrough technology of the year 2013 due to substantial benefit observed in clinical trials (1). In particular, the excitement refers to two therapeutic strategies, namely genetically engineered chimeric antigen receptor T cells and antibody mediated immune checkpoint inhibition (2, 3). Both strategies rely on stimulating and maintaining the patient's immune response to eliminate cancer cells.

Among the most advanced immune checkpoint inhibitors (ICIs) are nivolumab and pembrolizumab, which received marketing authorization for treatment of patients with a variety of cancer types in the US and the EU (4). These antibodies are directed against the receptor programmed cell death protein-1 (PD-1) that is expressed by many immune cells like activated T cells (5), B cells (6), natural killer cells (7), as well as monocytes and dendritic cells (DCs) (8, 9). Its ligand programmed cell death protein-1 ligand 1 (PD-L1) is expressed on a variety of cell types such as activated T cells (10) and DCs (11) as well as on a wide range of non-hematopoietic tissues like lung (12). Their physiological function is the maintenance of immunological homeostasis in the periphery during inflammation to prevent autoimmunity (8, 13). However, PD-L1 is also expressed on several tumor types as well as on tumor-infiltrating immune cells and PD-1 is upregulated on tumor-infiltrating lymphocytes (8, 14). Their interaction in the tumor microenvironment can inhibit local anti-tumor T cell responses and thereby promote immune escape of the cancer cells. Accordingly, antibodies such as nivolumab have been designed to block the interaction between PD-1 and PD-L1 in order to restore T cell activation and to prevent immune evasion of cancer cells (15).

While the clinical response in patients with advanced cancer is impressive, there are also downsides which include absence of response in some patients or toxicity induced by immune checkpoint modulation. The autoimmune-like toxicities are due to the systemic administration of the antibodies which do not only activate the immune response at sites of tumor lesions but also in healthy organs. A variety of frequently occurring so called immune-related adverse events (irAE) have been described including colitis, dermatitis as well as hepatitis. About 7–12% of patients receiving single-agent αPD-1/αPD-L1 monoclonal antibodies develop grade 3 or 4 irAEs (15, 16). Furthermore, rare but life-threatening irAEs such as pneumonitis have been described (15, 16). A key advancement in immune checkpoint blockade will therefore be the selective delivery of ICIs to tumor lesions. In preclinical tumor mouse models, the local, cell-mediated delivery of ICIs from GM-CSF-secreting tumor cells revealed potent anti-tumor responses without inducing autoimmunity-associated antibodies (17). Other researchers showed that the local administration of ICIs as Montanide emulsion close to the site of a subcutaneously growing tumor indeed reduced serum levels of the antibody by several orders of magnitude while retaining unimpaired tumor control (18). While the clinical applicability of this approach is limited to accessible tumor sites, this observation further supports the call for the tumor-specific delivery of ICIs.

Among the most promising vehicles for *in vivo* gene delivery are adeno-associated viral (AAV) vectors. AAV vectors are currently investigated in various clinical studies addressing genetic diseases such as hemophilia or inherited blindness (19, 20). Furthermore, the first marketed gene therapy medicinal product in the Western world was based on AAV vectors administered intramuscularly into patients suffering from a rare genetic disease in lipid metabolism (21). While diverse AAV serotypes show different preferences for certain tissues, they do not mediate selectivity for a distinct cell type defined by surface markers (22). Moreover, none of the natural serotypes show any preference for cancer cells. Therefore, different strategies for viral vector engineering have been developed to make vectors selective for the relevant cell type of a particular application. Among these is the alteration of entry receptor usage by incorporating high affinity ligands into the viral vector particles (23).

We have recently succeeded in redirecting receptor usage of AAV vectors (serotype 2) by incorporating designed ankyrin repeat proteins (DARPins) as ligands into the AAV capsid (24). The genetic fusion of the DARPin to AAV's capsid protein VP2 (viral protein 2) together with ablation of natural receptor binding by two point mutations in the capsid proteins resulted in AAV vectors that were specific for the target cell type. Among these receptor-targeted AAV vectors is a tumor-specific vector, which displays Her2/neu-specific DARPins on the capsid surface (Her2-AAV). Her2-AAV vectors enabled specific gene transfer in subcutaneous and disseminated Her2/neu^+^ positive tumor lesions in a xenograft tumor mouse model (25). When equipped with a cytotoxic gene, a single administration of Her2-AAV was sufficient to control tumor growth and to substantially prolong survival, while non-targeted AAV2 vectors even reduced survival compared to untreated animals due to liver toxicity (24, 25).

In the present study, we packaged the coding sequences of ICIs into tumor-targeted Her2/neu-specific AAV vectors. To evaluate the suitability of different antibody formats, two approaches were followed including self-complementary (sc) AAV vectors encoding murine αPD-1 in the scFv-Fc format and single-stranded (ss) AAV vectors encoding the full-length antibody nivolumab (human αPD-1). The respective AAV vectors were tested for *in vitro* and *in vivo* transgene expression as well as their anti-tumoral activity. The present study provides proof of concept that tumor-targeted AAV vectors can be used for the targeted delivery of ICIs to the site of tumor growth. Based on our findings, several strategies can now be followed to identify ideal therapeutic settings for this strategy.

## Materials and Methods

### Cell Culture

HEK293T, HT1080 (ATCC CCL-121), and MDA-MB-453 cells (ATCC HTB-131) were grown in Dulbecco's modified Eagle's medium (DMEM) supplemented with 10% fetal calf serum (FCS) and 2 mM L-glutamine. MOLT 4.8 and Raji cells (ATCC CCL-86) were grown in Roswell Park Memorial Institute (RPMI) 1640 medium supplemented with 10% FCS and 2 mM L-glutamine. RENCA-Her2/neu cells were kindly provided by Winfried Wels, Georg-Speyer-Haus Frankfurt (26) and cultured in RPMI supplemented with 10% FCS, 2 mM L-glutamine, and 0.48 mg/ml geneticin.

PD-1 expressing HT1080 cells (HT1080-PD-1) were derived from HT1080 cells (ATCC CCL-121). For this, the cDNA sequence of mouse PD-1 and a puromycin resistance gene were cloned into a lentiviral transfer vector resulting in the bicistronic plasmid pS-mPD-1-IRES-puro-W. HT1080 cells were transduced with VSV-G pseudotyped lentiviral vector delivering pS-mPD-1-puro-W and were selected using 10 μg/ml puromycin. For cultivation, HT1080-PD-1 cells were grown in DMEM supplemented with 10% FCS, 2 mM L-glutamine, and 10 μg/ml puromycin.

### Plasmids

The Her2-specific DARPin-VP2 fusion construct (pDARPin-VP2), the native (pRC), and HSPG binding site-mutated packaging construct (pRCmut) as well as the scAAV transfer plasmid encoding the reporter gene luciferase or GFP have been previously described (24). The codon-optimized coding sequence for murine αPD-1 was derived from the clone J43 and fused to the CH2-CH3 region of human IgG1 with an N-terminal HA tag and a C-terminal Myc-tag by gene synthesis (Eurofins) (disclosed in patent US 7,858,746 B2) and cloned into pCG. The coding sequence for nivolumab was generated (GeneArt) by coupling full-length heavy and light chain (Accession No. DB09035; codon-optimized) via a 2A self-cleaving peptide sequence derived from foot and mouth disease virus (F2A). Coding sequences were inserted into scAAV (for murine αPD-1) or ssAAV backbones (for nivolumab) via sticky end ligation using specific restriction sites that were PCR-annealed or included via primary gene synthesis, respectively. All constructs further contained a bovine growth hormone polyadenylation signal (BGH-PolyA) and were under the control of strong and ubiquitously active promoter [spleen focus-forming virus (SFFV), cytomegalovirus (CMV), chicken β-actin (CBA) or human elongation factor α (EF1α)]. ssAAV constructs additionally contained a woodchuck hepatitis virus posttranscriptional regulatory element (WPRE). Optimization of the AAV transfer plasmid encoding nivolumab were performed via exchange or insertion of sequences coding for the 2A self-cleaving protein from thosea asigna virus (T2A), a furin cleavage site (FurinCS) as well as a β-globin intron (BGI) or an intron derived from the minute virus of mice (MVM).

Oligonucleotide sequences are available upon request.

### Vector Particles

The adenovirus helper-free production of Her2-targeted AAV vectors and AAV2 was performed as previously described (25). A detailed protocol has been provided (27). Briefly, for the production of Her2-AAV, HEK-293T cells at 80–90% confluency were co-transfected with pXX6-80, pRCmut, pDARPin-VP2, and the vector transfer plasmid in a ratio of 3:1:1:1 using polyethyleneimine (PEI) as transfection reagent. For the production of AAV2 having an unmodified capsid, the plasmids pXX6-80, pRC, and the transfer plasmid are used in a ratio of 3:1:1. Cells were harvested 48 h after transfection, pelleted, and lysed via repeated freeze-thaw cycles. After benzonase (Sigma-Aldrich) treatment and clarification of the cell lysate, vectors were finally subjected to iodixanol density gradient centrifugation.

Nivolumab optimization was performed with small-scale (culture scale: single 150 mm diameter cell culture plate, cleared cell lysates) vector productions.

### Genomic Titer Determination via qPCR

Genomic DNA was isolated using the DNeasy Blood and Tissue Kit (Qiagen) according to the manufacturer's instructions. Subsequently, isolated DNA was subjected to TaqMan®-based quantitative PCR using the SensiFast^TM^ Probe No-ROX Kit (Bioline) and ITR-specific primers. Template DNA and a 10-fold serial dilution of standard plasmid DNA were amplified using 0.2 μM ITR forward, 0.68 μM ITR reverse, and 0.2 μM ITR probe. H_2_O served as negative control and the Light Cycler®480 PCR platform (Roche Life Science) was used for qPCR (polymerase activation: 95°C, 15 min, 4.4°C/s; 40x [denaturation: 95°C, 15 s, 4.4°C/s; annealing and extension: 60°C, 72 s, 2.2°C/s]; cooling: 40°C, 30 s, 1.5°C/s). Titers were calculated as genome copies per μl (gc/μl).

### *In vitro* Transduction and Analyses

#### Transduction

*In vitro* transgene expression upon transduction with Her2-AAV vectors and AAV2 was evaluated using cell lines that were either stably transfected to overexpress Her2/neu [renal adenocarcinoma (RENCA-Her2/neu)] or naturally express Her2/neu (MDA-MB-453). Transduction was performed in a 96-, 24- or 12-well plate format at a cell density of 8 × 103, 5 × 10^4^ or 8 × 10^4^ cells/well, respectively, using ≥450,000 genome copies/cell. Medium was exchanged after 24 h. For the detection and quantification of released antibodies by Western blot and nivolumab-ELISA, cells were washed in PBS before serum-free medium (virus production serum free medium, VPSFM, Thermo Fisher Scientific) was added. Four days after transduction, conditioned media were harvested, centrifuged at 300x g for 5–7 min to remove cell debris and stored at 4°C (or −80°C for long-term storage) for analysis.

#### Western Blot

For Western blot analyses, cleared supernatants were mixed with SDS loading buffer containing β-mercaptoethanol in the presence or absence of dithiothreitol (DTT) and subsequently subjected to SDS-PAGE (10% polyacrylamide gel). Separated proteins were transferred to nitrocellulose membranes using a semi-dry blot system (Biorad) and analyzed via immunostaining according to the manufacturer's instructions using either an antibody specific for binding to the HA-tag (mouse, clone 16B12; Abcam) combined with an HRP-conjugated secondary antibody (Dako) or an HRP-labeled antibody against human IgG (Sigma Aldrich) for the detection of AAV-encoded murine αPD-1 or nivolumab, respectively. Chemiluminescence detection on X-ray films was performed after treatment with Pierce^TM^ ECL Plus Western Blotting Substrate (Thermo Fisher Scientific).

#### Enzyme-Linked Immunosorbent Assay (ELISA)

Two different sandwich ELISA approaches were performed to evaluate concentrations of transgene expression, each being optimized for its specific application. Briefly, for the detection of AAV-encoded murine αPD-1, 96-well immunoplates (Nunc MAxiSorp, Thermo Fisher Scientific) were coated with 250 ng of an HA-specific antibody per well in PBS for 2–3 h (mouse, clone 16B12; Abcam), blocked (5% FCS, 0.05% Tween, PBS) and loaded with supernatant from transduced cells or organ lysates over night at 4°C. Bound AAV-encoded αPD-1 was finally caught by an HRP-conjugated antibody against human IgG (Sigma Aldrich) and visualized using 1-Step^TM^ Ultra TMB substrate (Thermo Fisher Scientific). Concentrated and protein affinity tag purified recombinant αPD-1 from transfected HEK293T cells was used to generate standard reference values.

For the detection of AAV-encoded nivolumab, plate coating was performed with 100 ng/well using recombinant human PD-1 protein (Sino Biological) in H_2_O overnight, while the remaining procedure was identical, only varying in secondary antibody concentration and incubation time (αhuFc-HRP 1:10,000 for 3 h and 1:500 for 2 h for murine αPD-1 and AAV-Nivolumab, respectively). Standard values were generated using the therapeutic drug Opdivo® (Bristol Myers Squibb).

#### Flow Cytometry

Specific target binding of AAV-encoded αPD-1 was evaluated using HT1080 cells genetically engineered to express murine PD-1 or MOLT 4.8 cells naturally expressing human PD-1. 5 × 10^4^ cells were incubated with 400 μl of cell culture supernatant from transduced cells for 1 h at 4°C. Specific binding of AAV-encoded αPD-1 was further detected via fluorophore-labeled antibodies against the human Fc region (Southern Biotech). To exclude unspecific binding, PD-1 negative cells (naïve HT1080 or Raji) were used as negative controls. Flow cytometry was performed on a MACSQuant Analyzer 10 (Miltenyi Biotec) and data were evaluated using FCS Express V6 (*De novo* Software).

#### Functionality Assay

To test the functionality of human αPD-1, a specifically designed assay was used based on the emission of luminescence upon reactivation of T cells due to PD-1 blockade (PD-1/PD-L1 Blockade Bioassay, Promega). The assay was performed according to the manufacturer's instructions and as described by others (28, 29). Briefly, target cells expressing PD-L1 and an engineered cell surface protein designed to activate cognate TCRs in an antigen-independent manner were incubated together with PD-1 effector T cells expressing PD-1 and a luciferase reporter driven by an NFAT response element (NFAT-RE). Upon interruption of PD-1/PD-L1 binding due to the addition of cell culture supernatant of AAV-transduced cells containing secreted αPD-1, TCR activation induces luciferase expression via the activation of NFAT that was detected by the addition of Bio-Glo^TM^ reagent. Luminescence was evaluated using a MicroLumat Plus microplate luminometer (EG & G Berthold).

### *In vivo* Analyses

#### Mice

For *in vivo* analysis of transgene expression and antitumoral activity, 8–10 week old BALB/c mice (Charles River) were engrafted with 5 × 10^6^ RENCA-Her2/neu tumor cells in 100 μl PBS via subcutaneous injection into the right flank under 2–3% isoflurane inhalation anesthesia. Tumor growth (mm3) (0.4 × length (mm) × [width (mm)]^2^), body weight, and overall health status of the animals were monitored at least twice per week throughout the study. Weight loss >20% and tumor ulceration were exclusion criteria, while tumor volume >800 mm3 was set as final endpoint. AAV2 and Her2-AAV vectors were administered intravenously into the tail vein or intratumorally under isoflurane inhalation when tumor growth reached distinct volumes (biodistribution studies: ~100 mm3; therapeutic study: ~50 mm3). Each vector dose contained 7 × 10^10^−3.6 × 10^11^ genome copies and was performed as single or double injection with 48 h interval. The chemotherapeutic agent Ixabepilone (Selleckchem) was dissolved in 1:9 ethanol:PBS (v/v) immediately prior to injection and administered at a concentration of 8 mg/kg in a maximal volume of 200 μl via intraperitoneal injection under isoflurane inhalation anesthesia at day 7 after the first vector administration.

#### *In vivo* Imaging

Vector biodistribution upon administration of AAV vectors encoding the reporter gene luciferase was monitored by *in vivo* imaging using the IVIS® Imaging System 200 (Caliper Life Sciences) as previously described (24). Briefly, intraperitoneal injection of 150 mg/kg D-Luciferin (Perkin Elmer) was performed and data were generated in anesthetized mice (isoflurane inhalation using XGI-8 Gas Anesthesia system) 10–20 min after substrate injection. Finally, mice were sacrificed, organs dissected, and imaged in order to evaluate origins of luciferase signal emission. Data analysis was performed with Live Image 4.3 software (Caliper Life Sciences).

#### Sample Collection and Preparation

At final time points (expression studies: day 7–10), mice were sacrificed via cervical dislocation under isoflurane inhalation anesthesia and blood sampling was performed via retro-orbital bleeding using glass capillaries. EDTA-coated tubes were used for collection, followed by serum recovery upon centrifugation at 2,000x g for 5 min. Extracted sera were stored at −80°C for analysis via ELISA as described in the previous section. Tumor and liver tissue were dissected and immediately snap-frozen in liquid nitrogen and stored at −80°C. At the day of preparation, tissues were thawed on ice, minced into small pieces and transferred to Lysing Matrix D Tubes (MP Biomedicals), followed by the addition of 2 or 1.25 μl Passive Lysis Buffer (Promega) supplemented with Protease Inhibitor Cocktail (Roche Applied Sciences) per milligram of tumor and liver tissue, respectively. Homogenization was performed using a Fast-Prep-24® instrument (MP Biochemicals) under continuous cooling for several cycles until tissues were completely dissociated. After an incubation period of 15 min at room temperature, tissue homogenates were transferred to fresh tubes and a two-step high speed centrifugation was performed (16,200 × g; 4°C; 20 and 10 min, respectively). Supernatants were stored at −80°C for analysis with another centrifugation (16,200 × g; 4°C; 10 min) immediately prior to ELISA performance to remove remaining precipitates (please refer to previous section for ELISA procedure).

## Results

### Generation of a Her2/neu-Targeted AAV Vector Encoding Murine αPD-1

**Figure 1:**
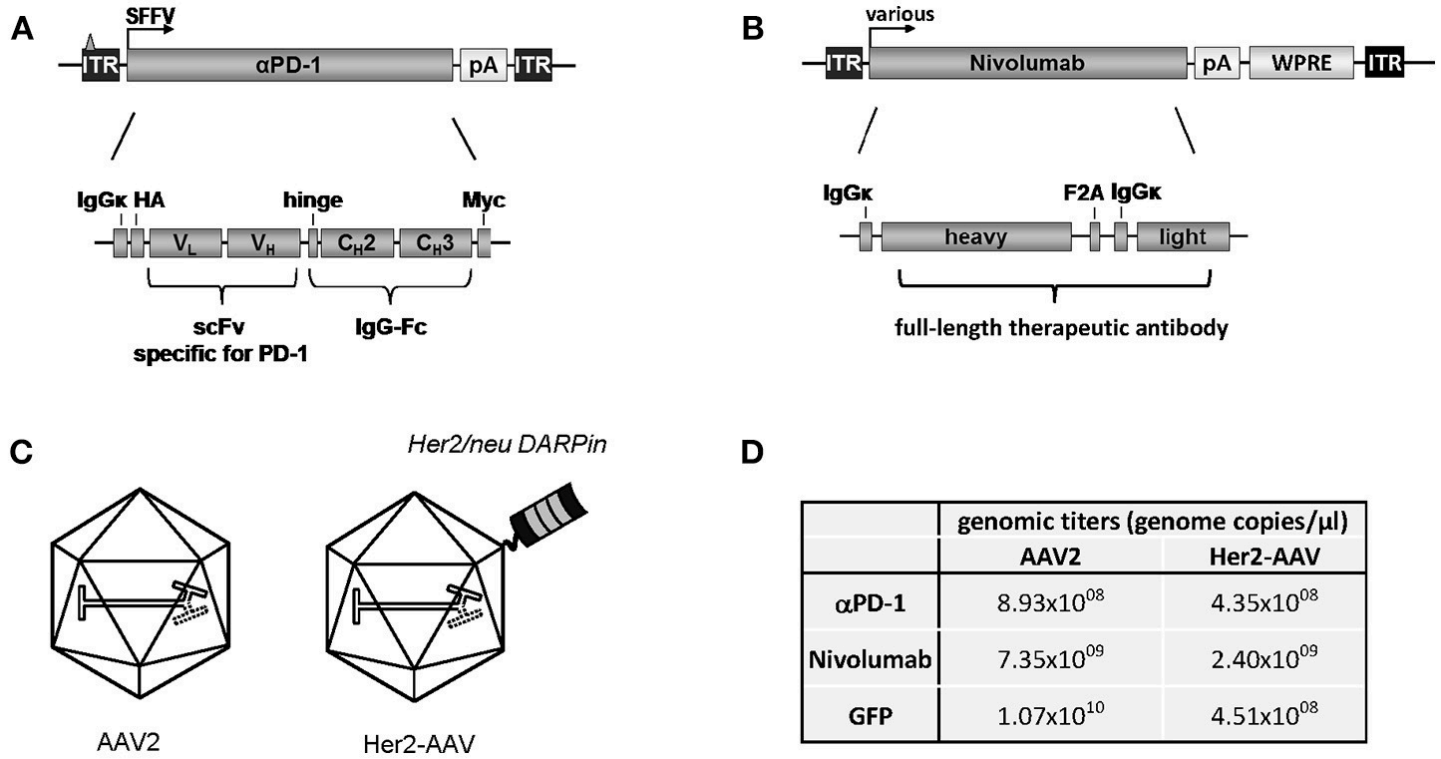
Cloning and characterization of AAV vectors encoding αPD-1. **(A)** Design of the transfer vector constructs encoding murine αPD-1 or **(B)** Nivolumab.ITR: inverted terminal repeat (with triangle representing the deleted terminal resolution site sequence in the scAAV construct); SFFV, spleen focus-forming virus; HA, hemagglutinin; V_H_, variable region of the heavy chain; V_L_, variable region of the light chain; C_H_, constant region of the heavy chain; scFv, single chain variable fragment; IgG, immunoglobulin G; Fc, fragment crystallizable; WPRE, woodchuck hepatitis virus posttranscriptional regulatory element; F2A: foot and mouth disease virus-derived self-cleaving peptide. **(C)** Schematic representation of AAV2 and Her2-AAV. The displayed DARPin providing Her2/neu specificity is depicted on the capsid surface. **(D)** Representative genomic titers of the indicated gradient-purified large-scale vector stocks.

Aiming at the tumor-targeted delivery of ICIs, we packaged the coding sequence for murine αPD-1 into Her2-AAV first. The αPD-1 construct was composed of a scFv specific for murine PD-1 fused to the Fc part of immunoglobulin G1 (IgG1) (Figure 1A). The corresponding open reading frame was cloned into an AAV2-derived self-complementary transfer vector plasmid under control of the spleen focus forming virus (SFFV) promoter. As control, a similar transfer vector was generated encoding only the constant region of IgG1 (IgG-Fc). The respective sequences were packaged into Her2-AAV or AAV2 with unmodified capsid (Figure 1C). Following purification of the vector particles via density gradient purification, genomic titers were determined, revealing values ranging from 4.35 × 10^8^ to 8.93 × 10^8^ genome copies per μl (Figure 1D). Of note, titers of these newly generated Her2-AAV vectors were in a similar range as those of well-established vectors encoding GFP (Figure 1D).

**Figure 2:**
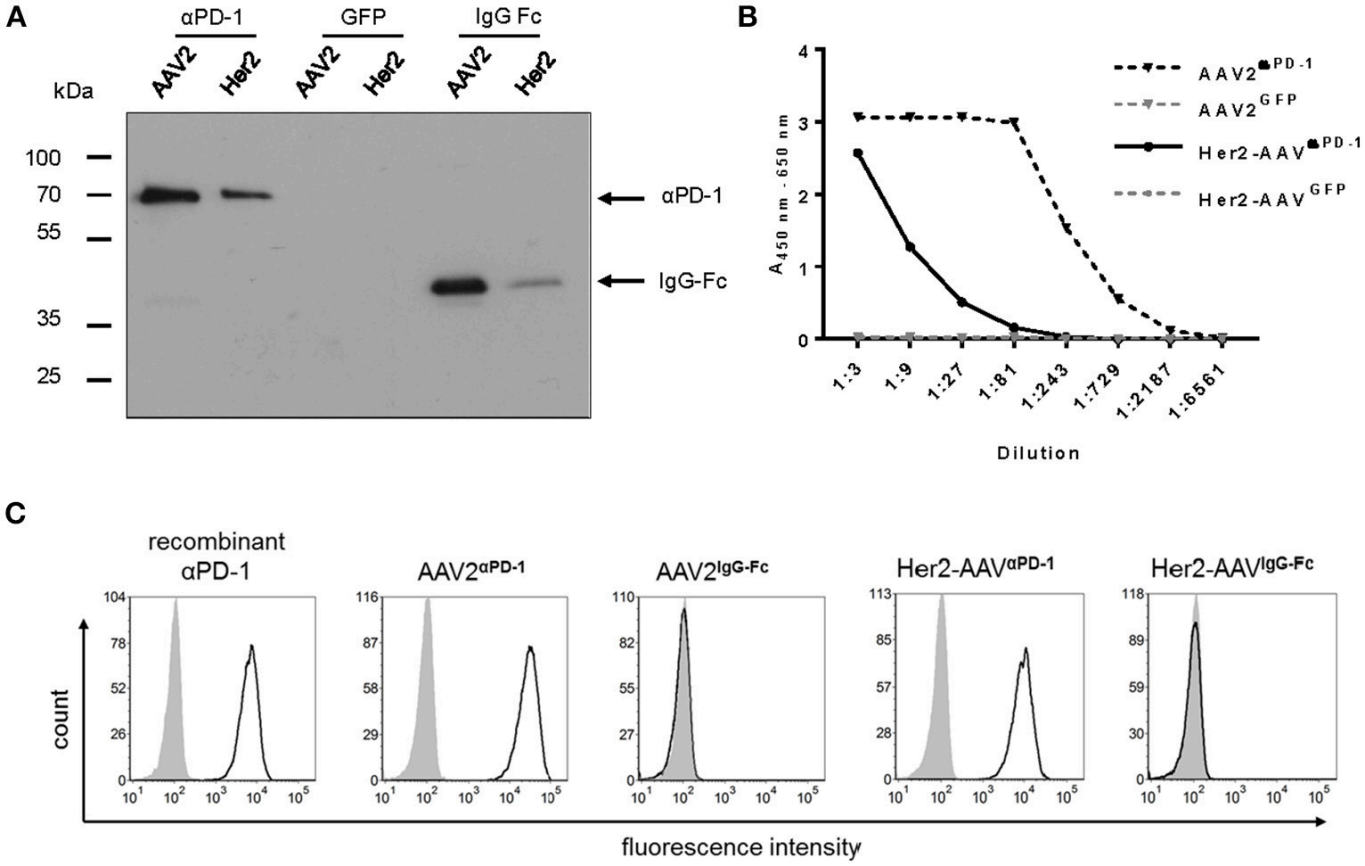
Characterization of AAV-encoded αPD-1. Four days after incubation of RENCA-Her2/neu cells with AAV2 or Her2-AAV vectors encoding αPD-1, IgG-Fc or GFP (450,000 genome copies/cell), cell culture supernatants were collected and transgene expression was analyzed. **(A)** Western blot analysis using an HA-specific antibody. **(B)** Sandwich ELISA with IgG-Fc- and HA-specific antibodies. **(C)** Specific antigen recognition of AAV encoded αPD-1 from cell culture supernatants of vector transduced cells and recombinant αPD-1 were assessed on PD-1 negative (gray shaded profiles) and PD-1 positive HT1080 cells (empty profiles, black line) by flow cytometry. The binding of αPD-1 was detected via an IgG-Fc-specific antibody. As further control, PD-1 positive and parental HT1080 cells were incubated with supernatant of AAV2^IgG−Fc^ transduced cells.

Transgene expression was assessed in cell culture supernatant of AAV2- and Her2-AAV-transduced renal cancer cells that had been genetically engineered to express the target receptor Her2/neu (RENCA-Her2/neu). Immunoblot analysis revealed signals at the expected size for αPD-1 and the control IgG-Fc (Figure 2A). Furthermore, enzyme-linked immunosorbent assay (ELISA) verified AAV-mediated αPD-1 expression and showed higher expression levels for AAV2- compared to Her2-AAV-transduced cells (Figure 2B). Quantification revealed concentrations of 12.6 ± 0.9 μg/ml for AAV^α*PD*−1^ (*n* = 3 technical replicates, mean ± SD) and 0.53 ± 0.04 μg/ml for Her2-AAV^α*PD*−1^ (*n* = 3 technical replicates, mean ± SD). αPD-1 collected from culture supernatant of AAV-transduced RENCA-Her2/neu cells specifically bound to PD-1 expressing HT1080 cells as revealed by flow cytometry analysis (Figure 2C). In summary, we successfully generated AAV vectors that led to efficient αPD-1 expression and target binding *in vitro*.

### Tumor Targeting by Her2-AAV in Immunocompetent Mice

**Figure 3:**
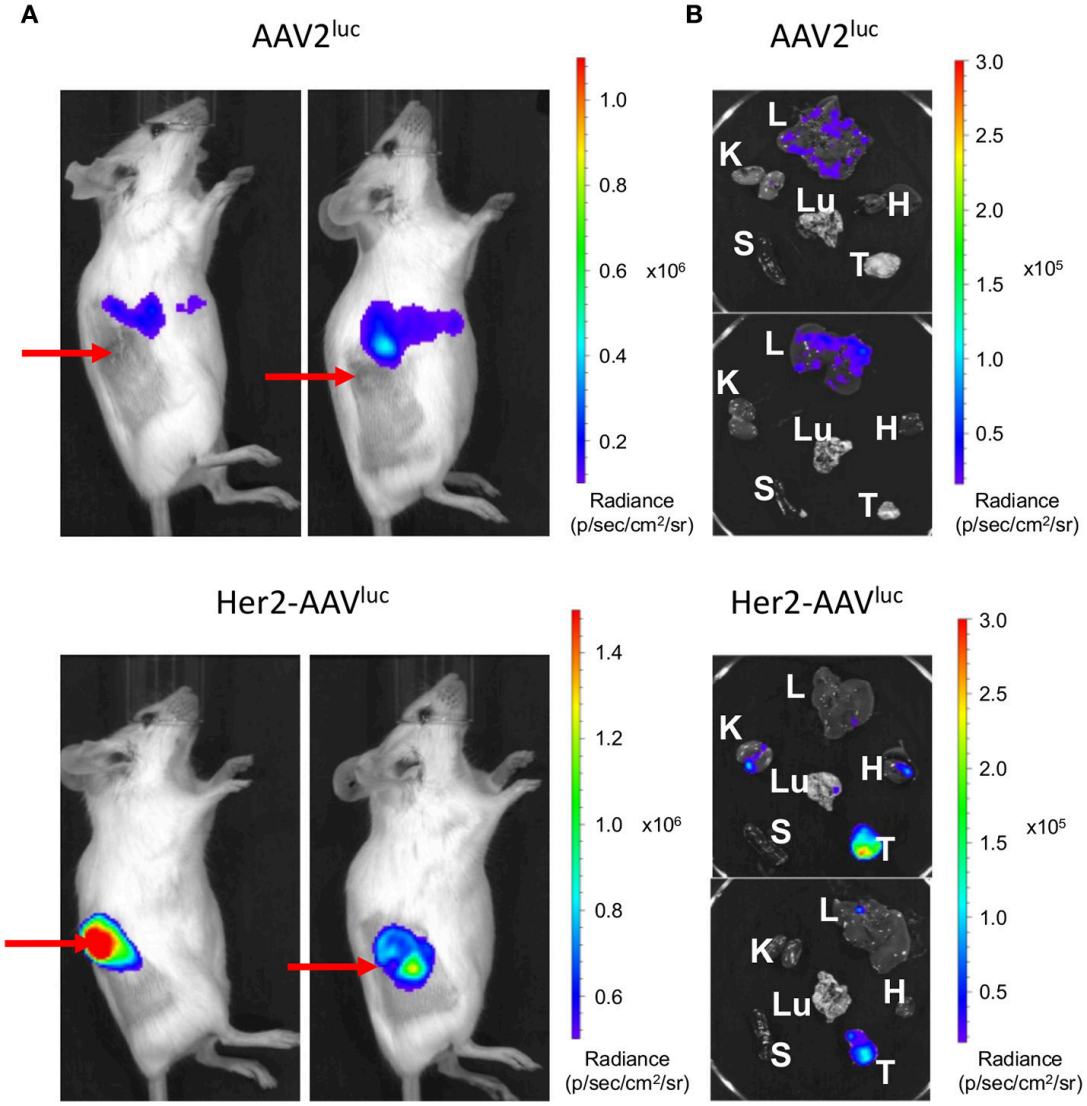
Tumor targeting by Her2-AAV in immunocompetent mice. 5 × 10^6^ RENCA-Her2/neu tumor cells were implanted subcutaneously into the flank of BALB/c mice. AAV targeting was analyzed by *in vivo* imaging one week after intravenous injection of 1 × 10^11^ genome copies of AAV2^luc^ or Her2-AAV^luc^. Luciferase signal intensity is expressed as photons/second/square centimeter/steradian (p/sec/cm^2^/sr). Luciferase expression was analyzed in living mice **(A)** and in isolated organs (L, liver; K, kidney; H, heart; Lu, lung; S, spleen; T, tumor) **(B)**. Red arrows point to subcutaneously growing tumors.

Tumor-specific gene transfer by Her2-AAV vectors has been previously demonstrated in immunodeficient xenograft tumor mouse models (24, 25). Since therapeutic effects by immune checkpoint blockade require a functional immune system, immunocompetent mice are obligatory for assessing Her2-AAV mediated delivery of ICIs. In this regard, BALB/c mice carrying subcutaneously growing RENCA-Her2/neu tumors were injected intravenously with Her2-AAV or AAV2 vectors transferring the luciferase reporter gene that can be monitored via *in vivo* imaging. Her2-AAV^luc^ vectors led to high luciferase signals in the tumor tissue, while AAV2-mediated luciferase expression was predominantly detectable in the upper abdominal region of the mice (Figure 3A). Final analyses of explanted organs identified the signal resulting from AAV2^luc^ injection to originate from liver tissue as expected based on the tropism of AAV2 (Figure 3B). These results demonstrate that Her2-AAVs can successfully target tumor tissue in an immunocompetent mouse model.

### Tumor-Targeted Delivery of Murine αPD-1 by Her2-AAV Vectors

**Figure 4:**
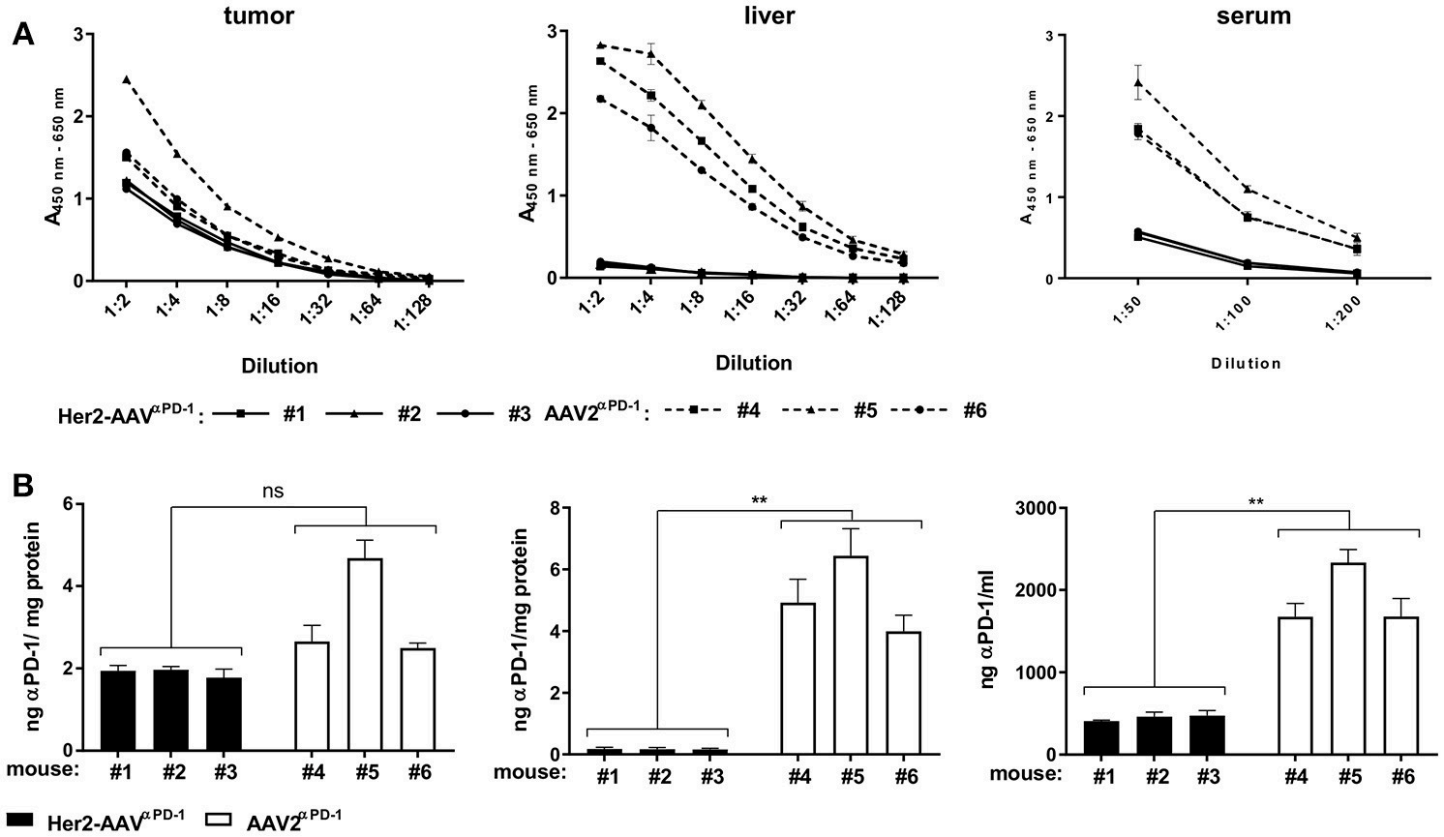
Tumor-targeted delivery of αPD-1 by Her2-AAV. RENCA-Her2/neu bearing BALB/c mice received a single intravenous injection of 1.1 × 10^11^ genome copies of AAV2^α*PD*−1^ (**A**: dashed lines, **B**: white bars), Her2-AAV^α*PD*−1^ (**A**: black lines, **B**: black bars) or PBS as mock control. One week after vector administration organs were explanted and sera taken. AAV mediated delivery of the immune checkpoint inhibitor in tumor and liver organ lysates as wells as in sera of the mice was analyzed by sandwich ELISA using IgG-Fc- and HA-specific antibodies. **(A)** Raw data obtained by ELISA. Values obtained with sera or organ lysates from PBS injected animals were subtracted from the ELISA signals of mice that had received AAV vectors. (*n* = 3 mice per organ and vector type are shwon; tumor: *n* = 1 per dilution, liver: *n* = 3 technical replicates per dilution, sera: *n* = 3–4 technical replicates per dilution; mean ± SD). **(B)** Amounts of αPD-1 present in tumor, liver, and serum. The amounts of αPD-1 in the organ lysates were normalized to the total protein yields. The quantity of αPD-1 in sera was calculated as ng/ml. (tumor: *n* = 3 technical replicates per mouse, liver: *n* = 9 technical replicates per mouse; serum: *n* = 5–7 technical replicates per mouse; mean ± SD). ^**^p < 0.01; ns, not significant by unpaired *t*-test (*n* = 3 mice per organ and vector type, numbers mean individual animal).

*In vivo* delivery of αPD-1 via AAV vectors was assessed in BALB/c mice with subcutaneous RENCA-Her2/neu tumors. Seven days after intravenous injection of Her2-AAV^α*PD*−1^ or AAV2^α*PD*−1^, levels of αPD-1 in tumor, liver, and serum of the mice were analyzed by ELISA. Raw data were quantified and normalized to the total protein yield of the organ extract (Figure 4). In the tumor tissue, comparable amounts of αPD-1 were detectable after injection of both vectors, ranging from 1.9 ± 0.11 ng αPD-1/mg protein (*n* = 3, mean ± SD) for Her2-AAV^α*PD*−1^ and 3.28 ± 1.22 ng αPD-1/mg protein (*n* = 3, mean ± SD) for AAV2^α*PD*−1^. Of note, the administration of AAV2^α*PD*−1^ revealed high levels in liver and serum of the mice [5.12 ± 1.24 ng αPD-1/mg protein (*n* = 3, mean ± SD) and 1,896 ± 378 ng/ml (*n* = 3, mean ± SD), respectively], while Her2-AAV^α*PD*−1^ injection resulted in significantly lower values [0.17 ± 0.01 ng αPD-1/mg protein (*n* = 3, mean ± SD) and 447.3 ± 36.7 ng/ml (*n* = 3, mean ± SD), respectively]. In summary, Her2-AAV mediated efficient and tumor-targeted delivery of murine αPD-1 in the scFv-Fc format in a syngeneic immunocompetent mouse model, thus enabling the evaluation of therapeutic implementations.

### Antitumoral Activity Upon Tumor-Targeted Delivery of Murine αPD-1

**Figure 5:**
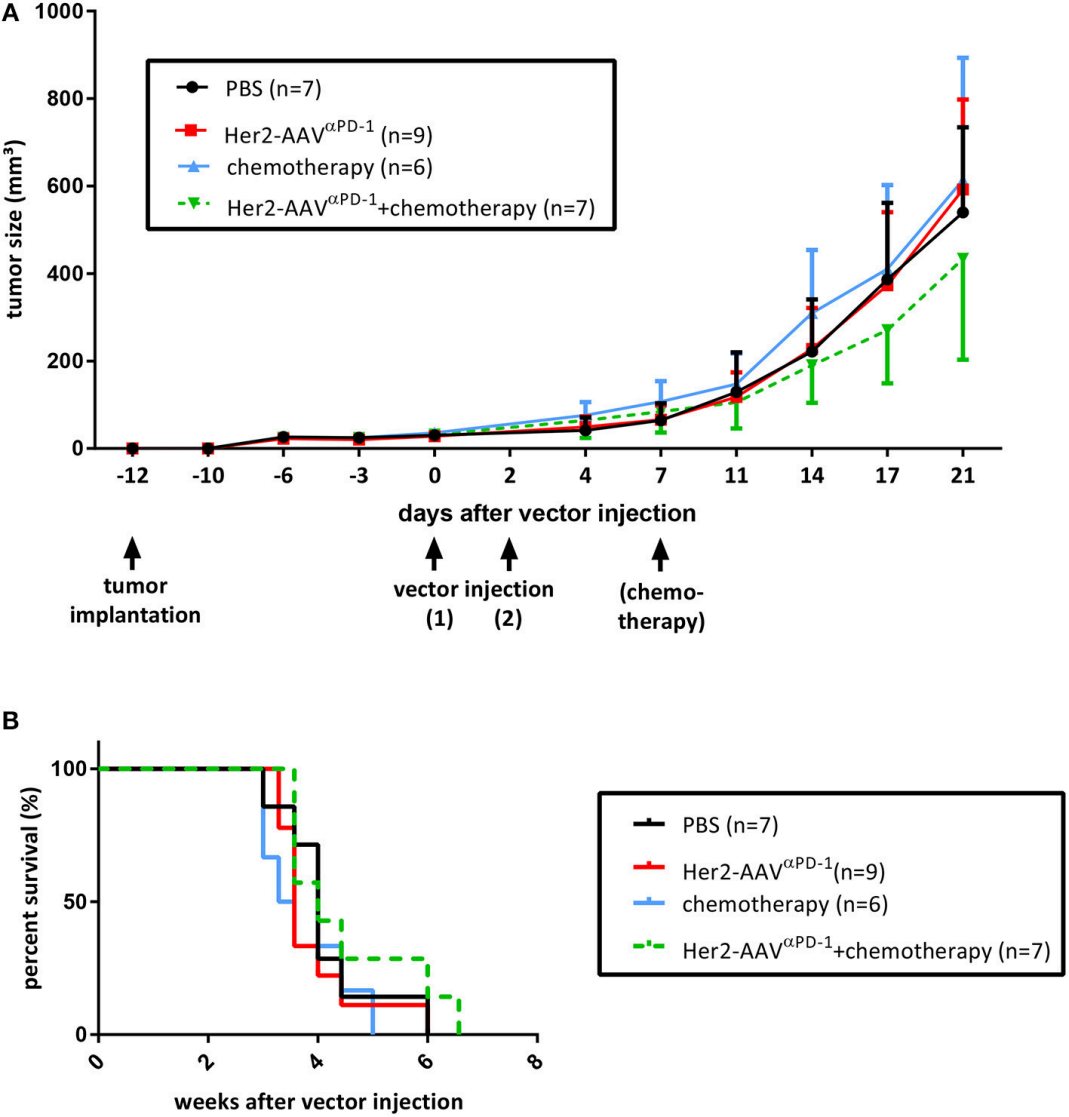
Therapeutic approach using Her2-AAV^αPD−1^. RENCA-Her2/neu bearing BALB/c mice received two intravenous injections of Her2-AAV^αPD−1^ (7 × 10^10^ genome copies each), either as a monotherapy (red lines) or in combination with the chemotherapeutical agent Ixabepilone (8 mg/kg; green dotted lines). Mice that received either chemotherapy alone (8 mg/kg; blue lines) or PBS (black lines) served as controls. Tumor size was monitored using the formula 0.4 × length × width^2^. Therapy was started when tumor sizes had reached 50 mm3 in average and mice were sacrificed when tumors were ≥800 mm3 in size. **(A)** Tumor growth curves with first sacrificed mouse selected as end-point (mean ± SD). **(B)** Kaplan-Meier survival curves.

In order to investigate the antitumoral activity of AAV-delivered αPD-1, an experimental setup in presence or absence of cytostatic agents was evaluated. BALB/c mice bearing subcutaneous RENCA-Her2/neu tumors were treated with Her2-AAV^α*PD*−1^ vectors, with or without the adjuvant administration of the chemotherapeutical agent Ixabepilone. Both groups as well as control mice (chemotherapy only, PBS) showed consistent tumor growth for at least 21 days after vector injection, while the mice that received combination therapy revealed slightly delayed progression (Figure 5A). These results were further reflected in survival analyses, showing no significant differences for all groups but minor survival advantage for mice that were treated with Her2-AAV^αPD−1^ vectors in combination with chemotherapy (Figure 5B). Although optimization for an effective treatment will be necessary, the administration of targeted AAV vectors encoding αPD-1 led to a marginal anti-tumoral activity when combined with chemotherapy.

### Generation of AAV Vectors Encoding Human αPD-1 (Nivolumab)

**Figure 6:**
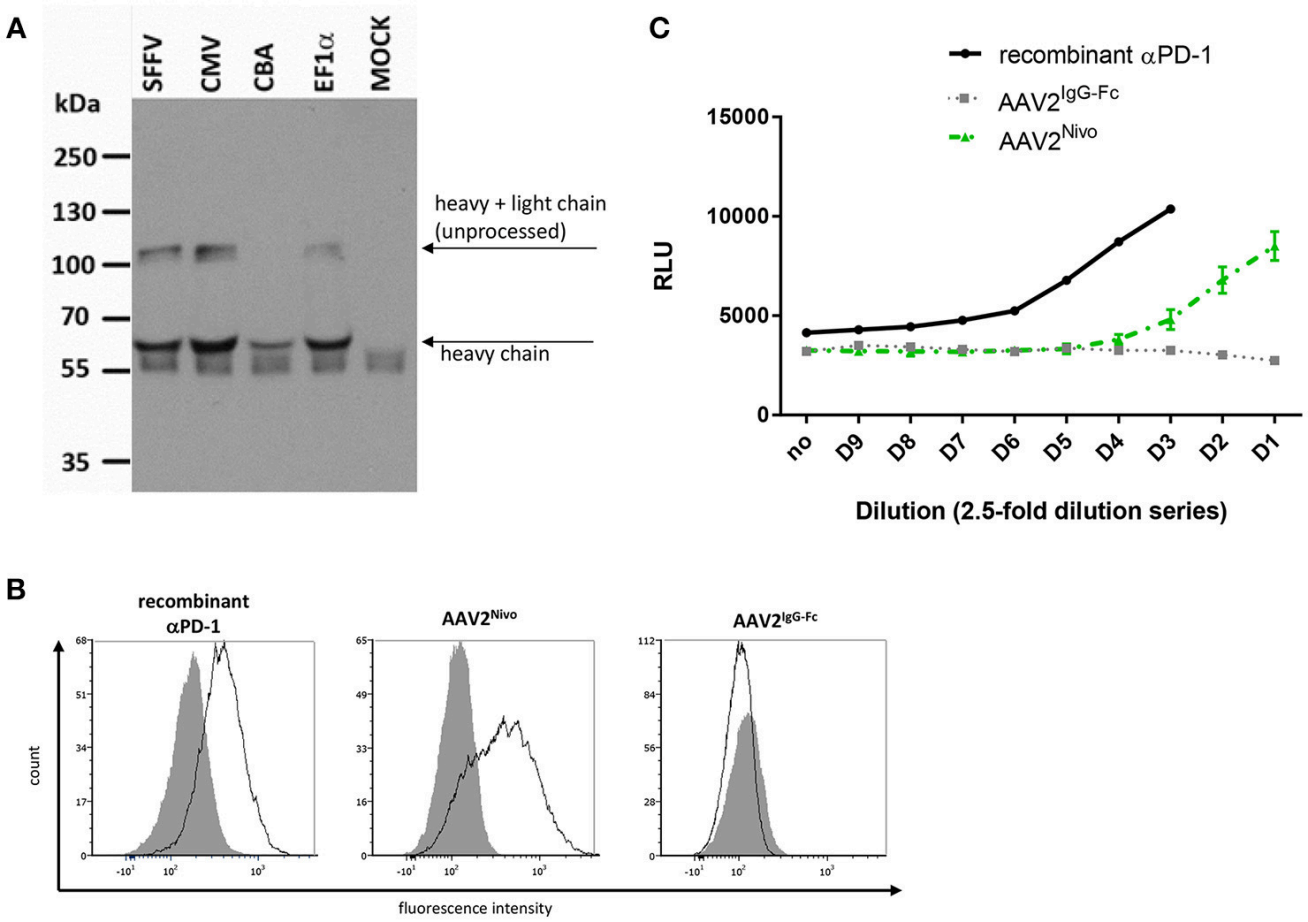
AAV encoded nivolumab specifically binds its target antigen. Four days after transduction of MDA-MB-453 cells with AAV2 vectors encoding nivolumab, cell culture supernatants were collected and transgene expression was analyzed. Vectors encoding for IgG-Fc (flow cytometry) or GFP (MOCK in Western blot) served as negative controls. **(A)** Western blot analysis using an IgG-Fc-specific antibody. Vector constructs (non-purified small-scale productions) with different promoters were used. SFFV, spleen focus-forming virus; CMV, cytomegalovirus; CBA, chicken β-actin; EF1α, human elongation factor 1α. **(B)** Specific antigen recognition of AAV-encoded nivolumab from cell culture supernatants of cells transduced with gradient-purified SFFV-driven vector stocks and a commercial recombinant αPD-1 were assessed on PD-1 negative (Raji; gray shaded profiles) and PD-1 positive cells (MOLT4.8; empty profiles, black line) by flow cytometry. The binding of nivolumab was detected via an IgG-Fc-specific antibody. **(C)** Assay to analyze functionality of AAV-encoded nivolumab (gradient-purified large-scale SFFV-driven vector stocks were used). Reactivation of T cells due to PD-1 blockade is correlated with increased luminescence signal indicated as relative light units (RLU). AAV2^Nivo^: green dashed line (*n* = 2 technical replicates; mean ± SD); AAV2^IgG−Fc^: gray dotted line (*n* = 1); recombinant αPD-1: black line (*n* = 1).

To assess if also a full-length IgG antibody specific for PD-1 can be delivered by AAV, AAV vectors were generated encoding the sequence of the authorized human αPD-1 antibody nivolumab. Due to the bigger packaging capacity required, we used AAV vectors in the single-stranded configuration, although these can lead to extenuated transgene expression values. The construct was designed through coupling the heavy and light chain via a 2A self-cleaving peptide sequence, while IgGκ leader sequences for secretion were fused to the N-terminal ends of both antibody chains (Figure 1B). Four different promoters were combined with the reading frame. Upon transduction of MDA-MB-453 cells, successful nivolumab expression was detected via Western blot analysis using an Fc-specific antibody (Figure 6A). An additional band migrating at ~100 kDa likely resulted from inefficient F2A processing (monomer of heavy and light chain). Signal intensities and thus yields of secreted nivolumab were similar for all constructs with exception of the CBA promoter mediating lower levels (Figure 6A). This was confirmed by ELISA (not shown). Hence, we finally continued using the SFFV promoter-driven AAV, termed AAV2^Nivo^, for further experiments (see Figure 1D for representative genomic titers). Specific binding of AAV2^Nivo^encoded nivolumab to PD-1^+^ MOLT4.8 cells was confirmed via flow cytometry analyses (Figure 6B). Moreover, its functional activity was evaluated by detecting T cell activity upon PD-1 blockade. In this assay, Jurkat cells expressing an NFAT-luciferase reporter intracellularly and human PD-1 on their surface were cocultivated with CHO cells expressing PD-L1, which inhibits TCR-mediated signaling and thus luminescence (28). With supernatant of tumor cells transduced with AAV2^Nivo^ (up to a dilution of 40-fold), luminescence became detectable whereas this was not the case when we used the control AAV encoding only the IgG-Fc part (Figure 6C). Thus, the supernatant of tumor cells transduced with AAV2^Nivo^ led to increased T cell activity confirming that AAV-encoded nivolumab activates the TCR downstream pathway in T cells.

### *In vivo* Delivery of Nivolumab via Her2-AAV Vectors

**Figure 7:**
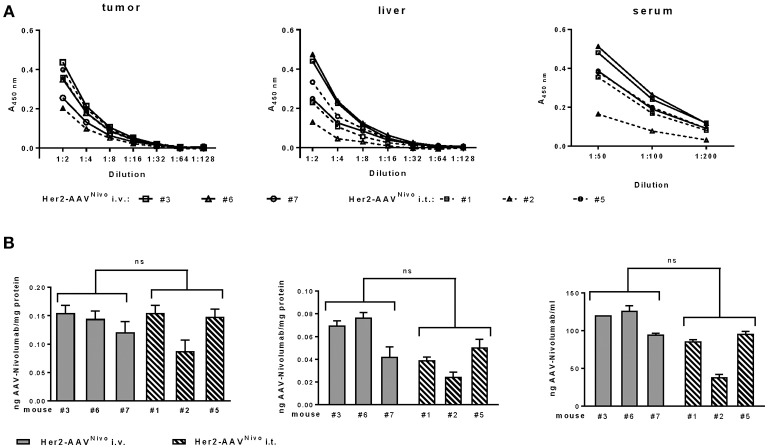
Tumor-targeted delivery of nivolumab by Her2-AAV. RENCA-Her2/neu bearing BALB/c mice received two injections of Her2-AAV^Nivo^ with 3.6 × 10^11^ genome copies each, either intravenously (**A**: black lines; **B**: gray bars) or intratumorally (**A**: dashed lines; **B**: striped bars). Mice that received only PBS served as mock control. After 10 days organs were explanted and sera taken. Nivolumab present in tumor and liver organ lysates as well as in sera of the mice was quantified by sandwich ELISA using plates coated with recombinant PD-1 and an IgG-Fc-specific detection antibody. **(A)** Raw data obtained by ELISA. Values obtained from PBS-injected animals were subtracted from the ELISA signals of mice that had received AAV vectors. **(B)** Amounts of nivolumab present in tumor, liver, and serum. Values were normalized to the total protein yields for the organ lysates and calculated as ng/ml for the sera [tumor: *n* = 2 technical replicates per mouse, liver: *n* = 3 technical replicates per mouse; serum: *n* = 3 technical replicates per mouse; mean ± SD; ns, not significant by unpaired *t*-test (*n* = 3 mice per organ and injection route; numbers mean individual animal)].

Having confirmed successful *in vitro* expression of functional, recombinant nivolumab by transduced cells, we next generated the corresponding Her2-AAV^Nivo^ vector and analyzed its potential for *in vivo* gene delivery of nivolumab. Her2-AAV^Nivo^ was injected intratumorally or intravenously into BALB/c mice bearing a subcutaneous RENCA-Her2/neu tumor. Final analyses were performed at day 10 after vector injection to compensate for a potentially slower expression kinetic of the single-stranded configuration in Her2-AAV^Nivo^ as compared to Her2-AAV^α*PD*−1^. Successful transgene expression in tumor tissue was confirmed upon intratumoral and intravenous Her2-AAV^Nivo^ injection. Overall, the amounts of nivolumab detected in tumor, liver, and serum were substantially lower than those detected for the murine αPD-1 antibody. In tumor, values for nivolumab ranged from 0.087 to 0.153 ng per mg total protein (Figure 7). For both administration routes, expression levels in the liver tissue were lower compared to the expression in the tumor. In liver and serum, the intravenous injection resulted in slightly but not significantly higher levels than the intratumoral vector administration (mean values: 0.062 vs. 0.038 ng/mg protein in liver and 112.91 vs. 72.53 ng/ml in serum of intravenous and intratumoral injected mice, respectively) (Figure 7). Although a confinement of transgene levels was observed, these data confirmed the tumor-targeted *in vivo* delivery of the full length antibody nivolumab via Her2-targeted AAV vectors.

### Improvement of Nivolumab Expression by Construct Design

**Figure 8:**
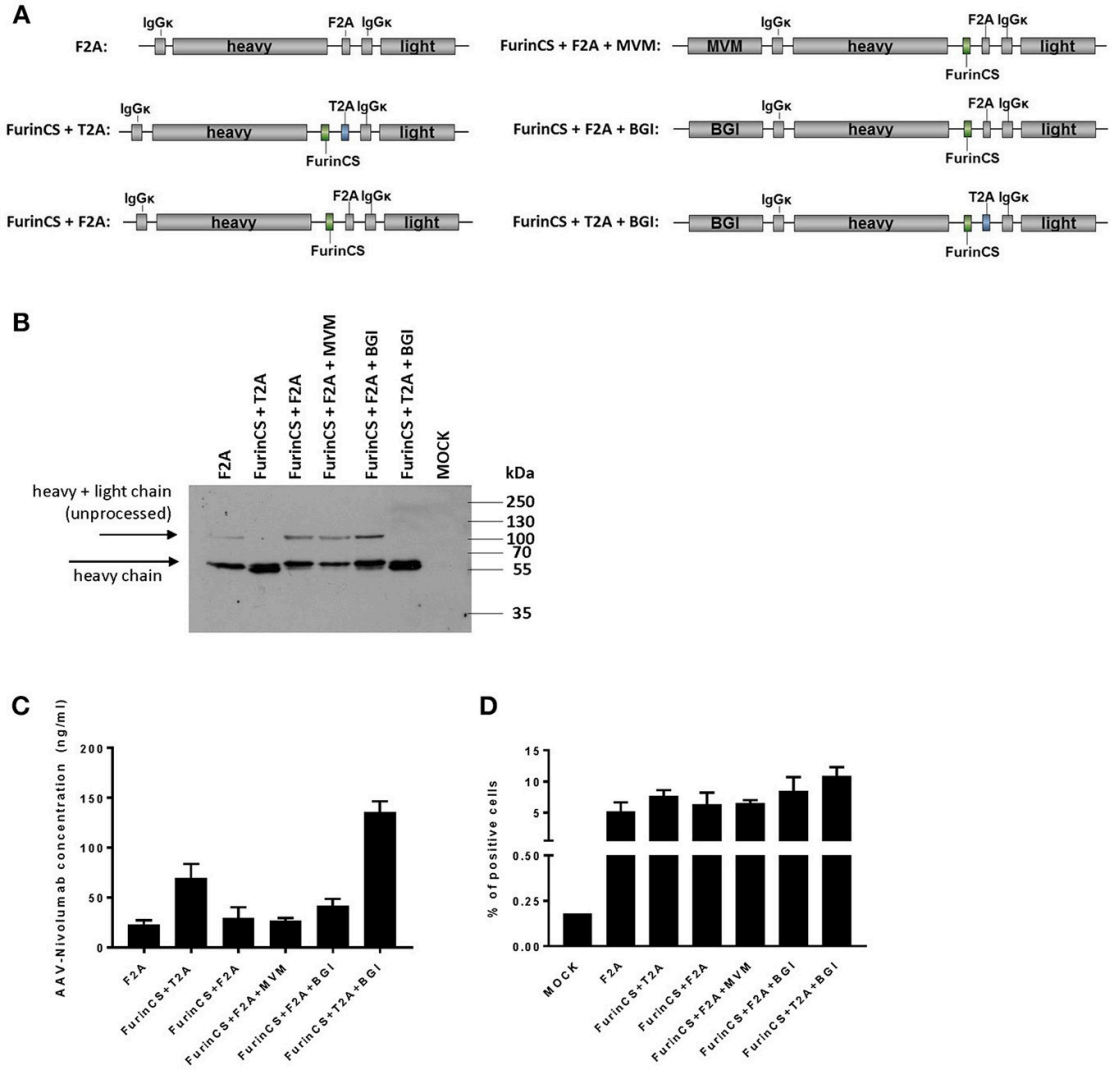
Optimization of nivolumab expression and processing. **(A)** Schematic drawings of optimized nivolumab expression constructs for AAV2 transfer vectors. All vector constructs were under control of the SFFV promoter. F2A, foot and mouth disease virus-derived self-cleaving protein sequence; T2A, thosea asigna virus-derived self-cleaving protein sequence; FurinCS, furin cleavage site; MVM, minute virus of mice intron; BGI, β-globin intron; IgGκ signal peptides are fused to the N-terminal ends of heavy and light chain. **(B)** Four days after transduction of MDA-MB-453 cells with small-scale preparations of AAV2 vector encoding the different constructs (500,000 genome copies/cell), cell culture supernatants were collected and analyzed by Western blot analysis using an IgG-Fc-specific antibody. AAV2^GFP^-transduced cells served as control (MOCK). **(C)** AAV-encoded nivolumab released into the vector transduced MDA-MB-453 cell culture supernatant was assessed by sandwich ELISA using plates coated with recombinant PD-1 and an IgG-Fc-specific detection antibody. **(D)** Specific antigen recognition was evaluated on PD-1 positive cells (MOLT4.8) by flow cytometry. The binding of nivolumab was detected via an IgG-Fc-specific antibody. MOLT4.8 cells were treated with supernatant of AAV2^IgG−Fc^ transduced cells (MOCK). All datasets in **(C,D)** *n* = 3 (mean ± SD).

It has been shown in previous studies that the inclusion of introns or alternative self-cleaving peptides in combination with a furin cleavage site (FurinCS) can enhance AAV-mediated expression of antibodies (30). Hence, in an attempt to enhance nivolumab expression levels, additional constructs were generated and tested for nivolumab expression (Figure 8A). Western blot analyses of supernatants from cells transduced with the corresponding AAV2 vectors revealed a specific band migrating at ~60 kDa and thus reflecting the immunoglobulin heavy chain for all constructs evaluated (Figure 8B). Of note, an additional band likely reflecting the precursor polypeptide encompassing heavy and light chain vanished, when the self-cleaving peptide was derived from thosea asigna virus (T2A) and combined with the FurinCS. In these samples, the bands corresponding to the nivolumab heavy chain were more intense and shifted to a slightly lower protein size, which is well in agreement with the activity of the furin cleavage site to remove remaining residues from the 2A processing. Quantification of nivolumab secreted by cells transduced with the different constructs revealed enhanced levels when the T2A-FurinsCS site was used. An about six-fold increased concentration of nivolumab was achieved upon combination with the β-globin intron (BGI) (Figure 8C). Finally, specific binding to PD-1 expressing MOLT4.8 cells was confirmed for nivolumab in supernatants of cells transduced with all vector variants, showing again a tendency for elevated binding when furin cleavage was combined with T2A and BGI (Figure 8D). Thus, we substantially improved the expression and processing of AAV-encoded nivolumab enabling the possibility to deliver ICIs as full-length IgG antibodies by this strategy.

## Discussion

The genetic delivery of therapeutic antibodies offers a number of advantages as compared to the direct administration of antibodies as protein (31). The use of AAV vectors for delivery can result in long-term antibody production upon a single injection thus potentially reducing manufacturing costs and treatment cycles. So far, AAV vectors have been mainly explored for the transfer of broadly neutralizing HIV antibodies toward the therapy of AIDS (31). In this setting, naturally available AAV serotypes deliver the antibody genes into liver or muscle tissue from where they are released into the blood stream resulting in persistent and high antibody serum levels. Here, we demonstrate that engineered AAV vectors can be used to deliver antibodies directed against immune checkpoints into tumor tissue thereby redirecting the biodistribution of ICIs from liver and serum to tumor tissue. While this is to our knowledge the first demonstration of tumor-targeted ICI delivery with a replication-deficient viral vector, oncolytic viruses encoding ICIs have recently been described.

Oncolytic measles virus and adenovirus have been engineered to encode the αPD-L1 scFv-Fc and αCLTA-4 scFv-Fc or the αCTLA-4 antibody, respectively (32, 33). Both these oncolytic viruses are replication-competent particles that lyse human tumor cells and have to be handled at biosafety level 2. While this property may enhance the invasion of tumor-infiltrating lymphocytes (TILs), they do not or only barely infect and replicate in mouse cells which makes preclinical models for these vector types challenging. The AAV vectors applied here can be handled at safety level 1. Additionally, there is now ample clinical experience with *in vivo* administrations of AAV particles that have been in focus of many clinical trials resulting in two products having received marketing authorization in the EU and US by now (34). This makes AAV vectors an ideal tool for the genetic delivery of ICIs as they can be much more easily applied for research and therapy than oncolytic viruses.

Our data demonstrate that Her2/neu-targeting results in a preferential expression of ICIs in tumor tissue over liver. Unmodified AAV2 vectors in contrast resulted in liver levels being roughly twice as high as those in tumor. Furthermore, serum levels were substantially higher in AAV2 treated than in Her2-AAV treated animals. Since tumors are connected to the blood circulation, release of tumor cell produced ICIs into the blood flow was expected. Interestingly, when we compared intravenous and intratumoral injection, serum levels tended to be higher via the systemic administration, while tumor levels were similar. This suggests that tumor cells directly in contact with blood vessels are more frequently hit upon systemic vector administration. These cells will then in turn be more effective in releasing the produced ICIs into the blood system.

While we provide evidence for the preferential expression of Her2-AAV encoded ICIs in tumor tissue, the antibody levels we detected were below those seen with the oncolytic adenoviruses mentioned above. However, these preclinical models differed in many aspects; among others we used immunocompetent mice whereas immunodeficient mice were used in context of oncolytic adenovirus (32). Another important aspect is the capability of tumor cells to express and secrete antibody molecules. It is reasonable that these cells are generally less efficient in releasing antibodies than plasma cells, but most likely this property differs between tumor entities and particular tumor cell types. Final conclusions can therefore only be drawn from side-by-side comparisons of the systems. Furthermore, it is currently unclear what serum and/or tissue levels are required for ICIs to exert their therapeutic activity. This will most likely differ between tumor mouse models and largely depend on the therapeutic strategy combined with the ICI. An intratumorally injected oncolytic poxvirus encoding the soluble form of PD-1 (vPD-1) exhibited less toxicity and was more effective than the combination therapy using intratumoral injection of unmodified virus and systemic application of αPD-1 antibodies (35). Notably, the ICI levels in vPD-1 injected tumor tissue and serum were in a similar range as those observed in our study suggesting that therapeutically relevant levels can be obtained with Her2-AAV vectors. In contrast to vPD-1, however, Her2-AAV is at least as effective in delivering the ICI upon systemic administration as upon local intratumoral injection. This is a major advantage when it comes to disseminated inaccessible tumor lesions.

For assessing antitumoral effects by immune checkpoint blockade therapy with Her2-AAV, immunocompetent models are mandatory. Therefore, RENCA-Her2/neu cells that are syngeneic to fully immunocompetent BALB/c mice were applied in the mouse model. These had been successfully used in other immunotherapy studies and were shown to be responsive to inhibition via the PD-1/PD-L1 axis (36–38). Yet, in our setting only a moderate tendency for reduced tumor progression was detectable. While we cannot exclude that the released ICI levels were too low, other reasons include but are not limited to a suboptimal PD-1 antibody format, a restricted amount of infiltrating T lymphocytes in the tumor tissue, and/or too ineffective chemotherapy or timescale issues regarding the coordination of tumor growth and therapeutic initiation. Furthermore, it should be pointed out that the human IgG1-derived Fc-part in αPD-1 on one hand facilitated easy detection of the vector encoded antibody by ELISA but can on the other hand trigger the murine antibody dependent cellular cytotoxicity (ADCC) system (39). This is especially relevant for PD-1-directed antibodies, since with these, ADCC directly compromises the activity of tumor-specific T lymphocytes (40). While all these issues may have contributed to the outcome of our preclinical study, it will be important to identify a tumor mouse model better suited to detect the therapeutic activity of ICIs delivered by Her2-AAV vectors. Also orthotopic tumor models should then be evaluated. Beyond that, there is room to improve ICI levels in tumor tissue not only by repeated vector injection, but also by removing DARPin-deficient AAV particles as recently described (25).

How flexible is the system? Her2-AAV vectors obviously can only be applied in Her2/neu-positive cancer. However, AAV vectors targeted against other tumor antigens including EpCAM (25), CD30 (41), and CD105 (42) have been described. A high-throughput based screening system for the identification of novel targeting ligands is available (42). Moreover, tumor–targeted AAV vectors selected from randomized capsid libraries and further enhanced for tumor selectivity by genetic elements can also be applied for this purpose (43, 44). Future studies will have to assess the suitability of each of these engineered AAV vectors for ICI delivery. Another important outcome of our study is that not only ICIs in the reduced size format but also full-length IgG antibodies as nivolumab can be delivered to tumor tissue by Her2-AAV vectors. Potential reasons for the lower *in vivo* levels obtained for nivolumab include, apart from the use of vectors in the single-stranded configuration, immune reactions against the human antibody, and enhanced diffusion of the AAV-encoded nivolumab into blood vessels due to low abundance of target cells (lymphocytes expressing human PD-1) in the selected tumor model. However, construct optimizations, especially with respect to processing efficiency as well as promoter and intron usage can substantially raise expression levels indicating room for further improvement. The AAV vectors described here are now available for straight-forward tumor-targeted ICI delivery allowing easy combination with other treatments like chemo- or radiotherapy. Also, combinations with CAR T cells or oncolytic viruses can be envisaged. Beyond therapeutic applications, the concept of targeting AAV vectors encoding ICIs to particular cell types may also be used to identify the relevant cell types for ICI production and to better understand their mode of action.

## Ethics Statement

This study was carried out in accordance with the recommendations of the German animal protection law. The protocol was approved by the Regierungspräsidium Darmstadt.

## Author Contributions

JR and JF planned and performed the experiments, analyzed data, and contributed to writing of the manuscript. CE and GU provided unique material and advised on their use. FT and JH evaluated data and contributed to experimental designs. CB initiated and supervised the project, acquired grants, planned experiments, and wrote the manuscript.

### Conflict of Interest Statement

The authors declare that the research was conducted in the absence of any commercial or financial relationships that could be construed as a potential conflict of interest.
